# Whole-scalp EEG mapping of somatosensory evoked potentials in macaque monkeys

**DOI:** 10.1007/s00429-014-0776-y

**Published:** 2014-05-04

**Authors:** Anne-Dominique Gindrat, Charles Quairiaux, Juliane Britz, Denis Brunet, Florian Lanz, Christoph M. Michel, Eric M. Rouiller

**Affiliations:** 1 Domain of Physiology, Department of Medicine, Faculty of Sciences and Fribourg Center for Cognition, University of Fribourg, Chemin du Musée 5, 1700 Fribourg, Switzerland; 2 Department of Fundamental Neurosciences, Faculty of Medicine, University of Geneva, Rue Michel-Servet 1, 1206 Geneva, Switzerland; 3 Functional Brain Mapping Laboratory, Departments of Clinical and Fundamental Neurosciences, University Medical School, Rue Michel-Servet 1, 1206 Geneva, Switzerland; 4 EEG Brain Mapping Core, Center for Biomedical Imaging (CIBM), University Hospital and University of Geneva, 1211 Geneva, Switzerland

**Keywords:** Craniotomy, Electrical neuroimaging, High-density EEG, LORETA inverse solution, Non-human primate, Sensorimotor cortex

## Abstract

High-density scalp EEG recordings are widely used to study whole-brain neuronal networks in humans non-invasively. Here, we validate EEG mapping of somatosensory evoked potentials (SSEPs) in macaque monkeys (*Macaca fascicularis*) for the long-term investigation of large-scale neuronal networks and their reorganisation after lesions requiring a craniotomy. SSEPs were acquired from 33 scalp electrodes in five adult anaesthetized animals after electrical median or tibial nerve stimulation. SSEP scalp potential maps were identified by cluster analysis and identified in individual recordings. A distributed, linear inverse solution was used to estimate the intracortical sources of the scalp potentials. SSEPs were characterised by a sequence of components with unique scalp topographies. Source analysis confirmed that median nerve SSEP component maps were in accordance with the somatotopic organisation of the sensorimotor cortex. Most importantly, SSEP recordings were stable both intra- and interindividually. We aim to apply this method to the study of recovery and reorganisation of large-scale neuronal networks following a focal cortical lesion requiring a craniotomy. As a prerequisite, the present study demonstrated that a 300-mm^2^ unilateral craniotomy over the sensorimotor cortex necessary to induce a cortical lesion, followed by bone flap repositioning, suture and gap plugging with calcium phosphate cement, did not induce major distortions of the SSEPs. In conclusion, SSEPs can be successfully and reproducibly recorded from high-density EEG caps in macaque monkeys before and after a craniotomy, opening new possibilities for the long-term follow-up of the cortical reorganisation of large-scale networks in macaque monkeys after a cortical lesion.

**Electronic supplementary material:**

The online version of this article (doi:10.1007/s00429-014-0776-y) contains supplementary material, which is available to authorized users.

## Introduction

Cortical plasticity promotes some functional recovery by allowing reorganisation of neuronal connections after a brain insult (e.g. Bütefisch 2004; Nudo 2006, 2007; Pascual-Leone et al. 2005). Different approaches such as intracortical microstimulation (ICMS) (Eisner-Janowicz et al. 2008; Liu and Rouiller 1999; Nudo and Milliken 1996; Rouiller et al. 1998; Rouiller and Olivier 2004; Wyss et al. 2013), laser speckle imaging (LSI) (Peuser et al. 2011) or magnetic resonance imaging (MRI) (Peuser et al. 2011) have been used to study cortical reorganisation accompanying functional recovery of manual dexterity after a motor cortex lesion in non-human primates. However, these techniques have major limitations: MRI has a great cost and a limited temporal resolution preventing time-sensitive assessment of the brain activity whereas ICMS and LSI are invasive methods limiting repetitive and long-term follow-up of functional reorganisation and their limited spatial coverage does not allow to investigate large-scale, whole-brain network recovery. In contrast, electroencephalographic (EEG) recordings of evoked potentials (EPs) are non-invasive, easy to use and to repeat, and offer high temporal resolution. The poor spatial resolution of conventional EEG techniques can be compensated using high-density scalp recordings and mapping analysis tools that render EEG a true brain mapping technique, i.e. providing spatiotemporal information on normal and pathologic brain functions (Jurcak et al. 2007; Michel and Murray 2012; Nunez 1993), as reported in humans (e.g. Hardmeier et al. 2013; Lascano et al. 2009, 2010; Lopez et al. 2011; Toepel et al. 2012; van de Wassenberg et al. 2008a, b, 2009) and in rodents (Megevand et al. 2008; Quairiaux et al. 2010). However, these tools have not yet been systematically used in monkeys. EEG recordings in monkeys were classically restricted to invasive electrocorticography or epidural recordings over a limited brain area (Allison et al. 1991a, b; Allison and Hume 1981; Arezzo et al. 1979, 1981; Chao et al. 2010; McCarthy et al. 1991) or were performed with surface electrodes along the stimulated afferent pathway (Hernandez-Godinez et al. 2011 (4 electrodes in *Macaca mulatta*)), on the skull (Reinhart et al. 2012 (14 electrodes in *Macaca radiata*); Tamura et al. 2013 (2 electrodes in *Macaca fuscata*)) or at the scalp with poor spatial resolution (Ferrari et al. 2012 (6 electrodes in newborn *M. mulatta*); Shimazu et al. 2000 (5 electrodes in *M. fuscata*); Ueno et al. 2008 (5 electrodes in *Pan troglodytes*)). To the best of our knowledge, only two studies involving whole-brain EEG recordings in monkeys have been reported, focusing on electrical source imaging of brainstem auditory evoked potentials (Fontanarosa et al. 2004, 32 electrodes in *M. mulatta*) and auditory event-related potentials (Gil-da-Costa et al. 2013, 22 electrodes in *M. mulatta*).

Motor areas and somatosensory areas are densely interconnected in primates and participate together to the motor control, forming the functional sensorimotor system (e.g. Huffman and Krubitzer 2001; Kaas 2004; Krakauer and Ghez 2000; Krubitzer and Disbrow 2005; Krubitzer and Kaas 1990; Shinoura et al. 2005; Stepniewska et al. 1993). The primary somatosensory cortex (S1) sends corticocortical inputs to the primary motor cortex (M1) (Ghosh and Porter 1988; Huerta and Pons 1990; Sloper 1973) and somatosensory corticospinal projections (Lemon 2008; Seo and Jang 2013), contributing to the control of voluntary movements (Murray and Keller 2011). After a lesion in caudal M1 in monkeys, the somatosensory system is affected in parallel with the motor control itself (Friel et al. 2005; Nudo et al. 2000) and in the same line, following a stroke, an increase of activity in S1 is associated with a better motor recovery in humans (Laible et al. 2012). It is therefore expected that, after a lesion in M1, S1 functions will also be affected in parallel with the motor control itself. Our long-term goal is to show that after a permanent lesion of the motor cortex, a rearrangement of connections, including also areas remote from the lesion, can be monitored at repetitive time points during the functional recovery using high-density EEG recordings of somatosensory evoked potentials (SSEPs).

To validate our approach of whole-scalp EEG mapping of SSEPs in macaque monkeys, a prerequisite is to demonstrate the intraindividual stability and interindividual reproducibility of SSEP signals. The present study intends therefore to demonstrate that SSEPs can be successfully and reproducibly recorded with good temporal and spatial resolution from a high-density EEG cap covering the entire skull in anaesthetized macaque monkeys. To this aim, we developed customised EEG caps with 33 channels for macaque monkeys. Median and tibial nerve SSEP recordings were regularly performed in five macaque monkeys to obtain stable baseline data. We assessed the intraindividual stability and interindividual reproducibility of the SSEPs with classical component analyses as well as topographical analysis tools as used in human studies. Furthermore, we evaluated for the first time the ability of SSEP source imaging to provide spatial information on brain somatosensory processing in macaque monkeys.

The experimental brain lesion of the motor cortex used in our non-human primate model requires a craniotomy (see e.g. Hamadjida et al. 2012; Kaeser et al. 2010, 2011; Liu and Rouiller 1999; Wyss et al. 2013). It is well-known from the literature that an opening in the skull may produce a strong distortion in the pattern of electrical fields recorded at the scalp due to a leakage of current through this hole and the surrounding skull (Brigo et al. 2011; Cobb et al. 1979; Cobb and Sears 1960; van Doorn and Cherian 2008). Therefore, the second goal of this study was to assess whether a craniotomy followed by bone flap replacement, suture and use of calcium phosphate cement to fill the gaps around the flap, would distort the SSEPs recorded from the scalp after surgery as compared to before surgery. This evaluation also has important implications for human EEG studies investigating recordings before and after surgery, e.g. in epileptic patients that are not seizure free after a surgery intended to remove the epileptic foci (see e.g. Jung et al. 2013; Moosa et al. 2013; Roulet-Perez et al. 2010; Sheybani et al. 2012; Simasathien et al. 2013).

## Materials and methods

### Macaque monkeys

Experiments were conducted on five adult macaque monkeys (*M. fascicularis*): three males (Mk-BB, Mk-DG, Mk-EN) and two females (Mk-AT, Mk-DI). Their age/weight ranges were 6 years/5.5 kg (Mk-BB), 9 years/8.5 kg (Mk-DG), 7–8 years/7.7–8.3 kg (Mk-EN), 7 years/3.3 kg (Mk-AT) and 8 years/3.4 kg (Mk-DI) at the time of the experiments. They were housed in the animal facility with one to three other congeners in a 45-m^3^ room (12 h light/dark cycle). The weight of the animals was checked daily. The animals were on no account food- or water-deprived (see e.g. Kaeser et al. 2010; Schmidlin et al. 2011). All procedures and animal care were conducted in accordance with the Guide for the Care and Use of Laboratory Animals (Committee for the Update of the Guide for the Care and Use of Laboratory Animals, National Research Council 2011) and were approved by local (Canton of Fribourg) and federal (Swiss) veterinary authorities. The present experiments were covered by the official authorisation numbers FR 22668, FR 18/10, FR 17/09, FR 156/08 and FR 22010. Experimental procedures were designed to minimise the animals’ pain and suffering.

### SSEP acquisitions

#### Anaesthesia and procedure

SSEP acquisitions were performed under sevoflurane anaesthesia (Sevorane^®^, Abbott) delivered with a mask by a cassette vaporiser inserted in an anaesthesia machine (ADU AS/3, Datex-Engström Division, Instrumentarium Corp., Helsinki, Finland). In case of low tolerance for the mask and also to decrease the level of stress in highly restless monkeys, a pre-anaesthesia with *S*-ketamine hydrochloride (Keta-S^®^, 60 mg/ml, Graeub AG, 5 mg/kg, im (intramuscular)) could be administrated as a first step. Data presented in this study were, however, obtained without this pre-anaesthesia. To induce a rapid gas anaesthesia, a bolus was first given at a concentration of 6.5 % of sevoflurane (1–2 l/min air; 1–2 l/min O_2_) for about 4–5 min, while the monkey sat in a Plexiglas^®^ primate chair (Schmidlin et al. 2011). Then, the concentration of sevoflurane was reduced and maintained at 2.5 % (0.3–1 l/min air; 0.3–1 l/min O_2_ for Mk-AT, Mk-BB and Mk-EN) or 3.5 % (0.7–1 l/min air; 0.7–1 l/min O_2_ for Mk-DG and Mk-DI) for the continuation of the experiment, suppressing the lid reflex. At that time, the monkey was placed in a metal tilted chair with the forearms on armrests and the hind legs laying horizontally on a platform in the prolongation of the chair. Monkey’s back and nape of the neck were maintained in an adequate position with a customised thermoplastic shell (Turbocast without perforation, Art.-nr 636025, FREY Orthopädie-Bedarf AG, Othmarsingen, Switzerland). The monkey’s head was shaved and washed vigorously with alcohol to eliminate fat secretion on the scalp. The EEG cap was then placed and maintained in the correct position using a chest strap. Body temperature was maintained by covering the animal with bubble wrap and single-use gloves filled with warm water. During the experiment, the level of anaesthesia was regularly evaluated by checking the lid reflex. Moreover, the electrocardiogram, the cardiac pulse frequency, the respiratory frequency, the expired CO_2_ and blood saturation rate in oxygen were continuously monitored with the anaesthesia machine.

Usually, a 30- to 50-min period was necessary between the induction of the anaesthesia and the first recordings to set up the EEG cap (see below) and to ensure equilibration of the anaesthetic concentration.

At the end of the recordings, the sevoflurane delivery was stopped and a mixture of O_2_ and air was delivered via the mask to the monkey. All SSEP recordings were performed in a Faraday cage room. The entire experimental session typically lasted about 2–3 h.

#### Peripheral nerve stimulation

An electrical pulse stimulation was delivered to the median nerve at the wrist or to the tibial nerve at the ankle, successively on both sides through a surface stimulator (barrette with 2 electrodes, 1 cm apart) attached around the corresponding limb with a Velcro^®^ strip. A silver-impregnated conductive Velcro^®^ ground electrode (model F-E10SG1, Grass Instruments Division, Astro-Med, Inc., West Warwick, RI, USA) was placed around the stimulated limb, proximally to the stimulation site. The regions where the stimulator and the ground electrode were applied were shaved and cleaned with alcohol and the electrodes of the stimulator were moistened with saline solution. Stimuli consisted of monophasic square wave electrical pulses of 400-μs duration delivered via an isolation unit (Stimulus isolator A365R, World Precision Instruments, Sarasota, USA) every 2 s (0.5 Hz) for periods of 3 min (corresponding to a total of about 90 stimuli delivered at each of the 4 stimulation sites). Stimulation intensities corresponded to the visual threshold of the motor response of the muscles innervated by the stimulated nerve (between 0.55 and 4.75 mA at the wrist and between 0.32 and 2.6 mA at the ankle, depending on the animal’s corpulence), i.e. eliciting a small twitch of the thumb after median nerve stimulation and a plantar flexion of the toes after tibial nerve stimulation. The visual motor threshold was used as an indicator of the stimulation intensity because it is a simple and position-independent criterion to visualise the effectiveness of the electrical stimulation. Moreover, this technique is widely used in clinics, e.g. to reliably localise a nerve or a plexus during peripheral nerve blockade (Tsui 2007) or to evaluate curarisation level in anaesthetised patients (Baurain et al. 1998) or animals (Martin-Flores et al. 2008). To reduce the likelihood of anodal block, the cathode of the stimulator was placed proximally and the anode more distally on the stimulated limb (Cruccu et al. 2008).

#### Scalp SSEP recording

**Fig. 1:**
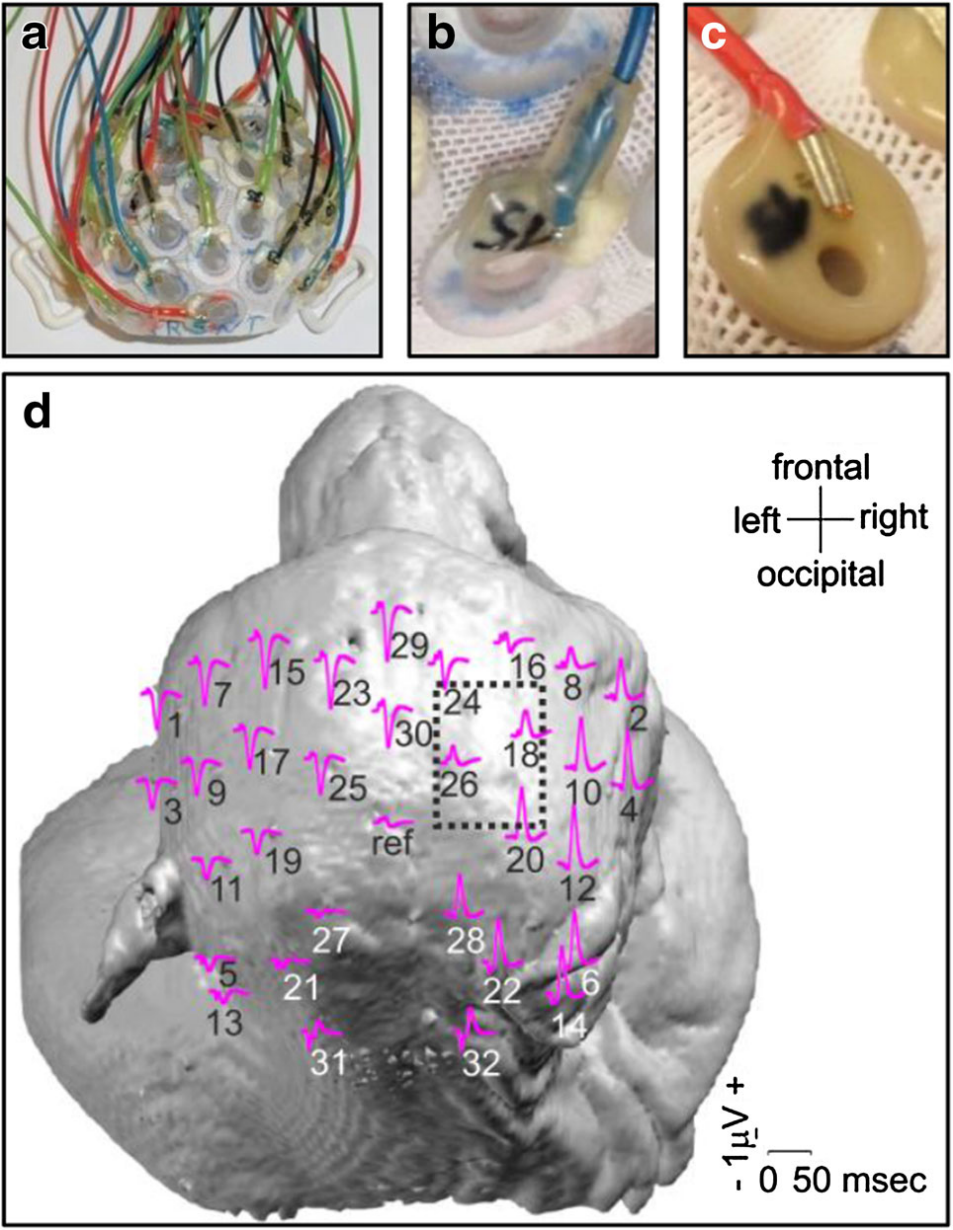
SSEP acquisitions and craniotomy. **a** View of Mk-EN’s EEG cap from the top (frontal: down, occipital: up, right side: *left*, left side: *right*). **b** Detail of an electrode of Mk-EN’s cap. The electrode is mounted on a 14-mm circular support which is inserted in the cap. **c** Detail of an electrode of Mk-AT, Mk-BB and MK-DI’s cap. The electrode is fixed on the cap via a 9.5-mm circular glue support. **d** Location of the 33 electrodes and their waveform after left median nerve SSEPs (GA of 9 recordings), projected on Mk-EN’s MRI head surface viewed from above (frontal: *up*, occipital: *down*, right side: *right*, left side: *left*). Interval: 0.0 to 50.0 ms post-stimulus. The perimeter of the bone flap of the craniotomy is indicated by *dotted lines*. *ref* reference electrode

Recordings were performed at the scalp with a customised EEG cap made of synthetic elastic tissue (EASYCAP GmbH, Herrsching, Germany) with slits for the ears and 32 sintered Ag/AgCl electrodes (EASYCAP, Catalogue-Nr. B12-HS-200) (Fig. 1a) in reference to a vertex electrode. The EEG was grounded at an electrode just left to the reference one. Electrodes were 2 mm in diameter and embedded on a circular support 14 mm in diameter for Mk-DG and Mk-EN’s cap (Fig. 1b) and 9.5 mm in diameter for Mk-AT, Mk-BB and Mk-DI’s caps (Fig. 1c). Electrodes were inserted in the cap in a symmetrical and regular manner between both hemispheres, based on the International 10–10 System (American Clinical Neurophysiology Society 2006) to cover the whole scalp (Fig. 1d). The montage included 2 midline sites and 15 sites over each hemisphere. The inter-electrode distance was between 1.5 and 2 cm for Mk-DG and Mk-EN’s cap and between 1 and 2 cm for the other caps.

The EEG was recorded using a BrainAmp DC amplifier (Brain Products GmbH, Gilching, Germany) with a band-pass filter between 0.1 Hz and 1 kHz and sampled at 5 kHz, with a resolution of 0.1 μV and 16 bit A/D conversion. Signals were displayed online and stored on hard drive using a conventional human EEG software (BrainVision professional Recorder 1.20, Brain Products GmbH, Gilching, Germany). Impedance minimisation was obtained with a high-chloride, abrasive electrolyte gel (Abralyt HiCl, EASY CAP). The impedances were kept below 5 kΩ.

### SSEP analysis

#### SSEP data averaging

Data analysis was performed using the Cartool software developed by Denis Brunet (Geneva University Hospital and Medical School, Geneva, Switzerland; https://sites.google.com/site/fbmlab/cartool, Brunet et al. 2011).

Prior to averaging, the EEG was filtered offline between 8 and 300 Hz, and the DC component (0 Hz) was removed. When the signals were contaminated with 50-Hz noise, an additional 50-Hz notch filter was used. Signals were re-referenced offline against the average reference and the reference electrode became therefore a 33rd electrode. Before averaging, responses to each stimulus were visually selected offline to ensure that they were not contaminated by noise in addition to a threshold criterion of 100 μV. All SSEPs were finally obtained by averaging about 80 sweeps. Data were baseline corrected, based on the −80 to −30 ms pre-stimulus period. For Mk-EN, several SSEP grand averages (GAs) were calculated based on different recordings made at days to weeks intervals before and after a 15 × 20-mm^2^ craniotomy performed over the right parietal bone (see below “Surgery and craniotomy”).

#### Classical component analysis

The effect of a craniotomy on EEG signals recorded at the scalp was first assessed by statistically comparing at each time frame the amplitude of left median nerve SSEP signal recorded at each electrode between 4 pre-craniotomy sessions and 4 post-craniotomy sessions. A two two-tailed unpaired *t* test with Bonferroni correction for the number of electrodes was performed in Cartool with a *p* value threshold at 0.01.

Moreover, to assess the effects of the craniotomy on SSEP signals and at the same time the stability of recordings, we also performed classical component analysis on the global field power (GFP) waveform and at two selected electrodes: electrode 32 (e32), located in right occipital region presumably over the right half of the brainstem, and electrode 12 (e12) presumably located over the sensorimotor cortex of the right hemisphere (see Fig. 1d for precise location of the electrodes). The arrival of the afferent volleys at the brainstem (brainstem component*)* and the main cortical component were characterised both on the GFP waveform, on e32 for the former and on e12 for the latter. Both components measured at peak were analysed in terms of absolute amplitude and latency from the stimulation onset on the 4 pre-craniotomy sessions and on the 4 post-craniotomy sessions. Pre- and post-craniotomy values were compared using unpaired *t* tests with SigmaPlot 12.0 (*p* value threshold at 0.01).

#### Identification of SSEP maps by cluster analysis

The spatiotemporal dynamics of the SSEPs can be represented by maps of the scalp potentials, i.e. a succession of non-overlapping periods of stable scalp voltage topographies of variable duration and intensities and separated by sharp transitions. Such SSEP component maps are thought to represent the different processing steps in the brain activity evoked by a stimulus (Brandeis and Lehmann 1986; Lehmann et al. 2009; Michel et al. 2009; Pascual-Marqui et al. 1995). These maps represent the voltage distribution on the scalp produced by all concurrently active intracranial generators during this processing step (Koenig et al. 2013; Michel and Murray 2012): different scalp topographies necessarily result from different generators (Helmholtz 1853; Michel et al. 2004; Pascual-Marqui et al. 1995; Vaughan 1982).

Topographical analyses of SSEPs have many advantages over the classical component analysis of SSEPs, namely SSEP maps are reference–independent and are not limited by a priori time periods and components of interest in a subset of electrodes (Geselowitz 1998; Michel et al. 2004; Murray et al. 2008).

To determine the most important stable maps optimally summarising the data, we applied a K-means clustering algorithm (Murray et al. 2009; Pascual-Marqui et al. 1995) to the GAs from 6- to 50-ms post-stimulus for median nerve SSEPs and from 10.8- to 60-ms post-stimulus for tibial nerve SSEPs. Clusters shorter than 0.4 ms (2 time frames) were excluded and associated with the preceding or the following one, depending on which they correlated better with. Clusters with a correlation coefficient >92 % were merged together. The optimal number of clusters was determined using either the Krzanowski–Lai criterion (Krzanowski and Lai 1988) or the cross-validation criterion (Brunet et al. 2011).

Potential values were averaged during each cluster at each electrode and interpolated with Delaunay triangulation for graphical representations of mean SSEP maps. For further details about the whole segmentation process, see Brunet et al. (2011) and Cartool Reference Guide (Brunet 1996).

#### Statistical analyses on SSEP component maps

To determine in how far the templates (or maps) identified by the cluster analysis are represented in the data of each individual recording session, we computed a strength-independent spatial correlation (SC) between the templates identified in the GA cluster analysis and the EPs of each individual recording session (fitting) (Megevand et al. 2008). The SC measures the topographical similarity between two maps:

$$\text{SC} = \frac{{\sum_{i = 1}^{n} {(u_{i} \cdot v_{i} )} }}{{\sqrt {\sum_{i = 1}^{n} {u_{i}^{2} } } \cdot \sqrt {\sum_{i = 1}^{n} {v_{i}^{2} } } }},$$

where *n* is the number of electrodes and *u*_*i*_ and *v*_*i*_ are the voltages against the average reference at electrode *i* for the two maps (Lehmann and Skrandies 1980). No SC between the template and the individual data results in SC = 0, whereas a perfect SC between them yields SC = 1. This fitting process assigns at each time frame and for each individual recording the component map obtained by cluster analysis having the highest SC. Segments shorter than or equal to 0.4 ms were rejected. No smoothing was applied to the data. Eight different topographical parameters were then computed for each component map in each individual recording: latency at first onset, duration, global explained variance (GEV), latency at best SC, mean SC, maximum of GFP, latency at maximum of GFP and mean GFP. The GEV is the sum of the explained variances weighted by the GFP at each time. For further details about these parameters, see Brunet et al. (2011), Koenig and Gianotti (2009) and Cartool Reference Guide (Brunet 1996).

The intraindividual stability of SSEPs was tested across 9 recording sessions. Unpaired *t* tests or Mann–Whitney *U* rank sum tests when normality tests failed were performed with SigmaPlot 12.0 to compare the latency at first onset and the latency at best SC from each individual recording session between pairs of successive component maps obtained after median nerve stimulation and tibial nerve stimulation (the *p* value threshold at 0.01 was adapted using Bonferroni correction for the number of electrodes). The total GEV for each recording session was also computed by adding the GEVs obtained from each template map, and these values were then averaged across the 9 recording sessions.

Moreover, two-tailed unpaired *t* tests with Bonferroni correction for the number of electrodes were performed with Cartool with a *p* value threshold at 0.01 to compare the 8 topographical parameters of the fitting before and after the craniotomy.

### SSEP source estimation method

#### MRI acquisition, electrode position reconstruction and lead field model

Because any voltage topography recorded at the scalp can be generated in principle by an infinite number of different source combinations within the brain, no unambiguous statement about which brain areas contribute to what extent to the EPs can be made on the basis of scalp EEG data alone. To estimate the electrical source activity, we used a low-resolution electromagnetic tomography (LORETA) distributed, linear inverse solution based on the estimation of current density (CD) distribution in the whole brain (Pascual-Marqui et al. 1994, 2009) combined with a lead field model (or forward solution model) based on the individual CT scan and MRI of Mk-EN.

For the MRI acquisition, the monkey Mk-EN was first sedated with an im injection of ketamine hydrochloride (Ketasol 100^®^, 100 mg/ml, Graeub AG, 10 mg/kg) and midazolam hydrochloride (Dormicum^®^, 5 mg/ml, Roche Pharma SA, 0.1 mg/kg), allowing to transport the animal from the animal facility of the University to the HFR Hôpital cantonal of Fribourg. The transport by car was approved by local (Canton of Fribourg) veterinary authorities. The MRI investigations were conducted according to guidelines established by the Hospital’s authorities.

Once in the MRI anteroom at the Hospital, the EEG cap was positioned on the animal’s head and a small spot of EEG paste (high-chloride electrolyte gel Lectron III-10, EASY CAP) was put at each electrode location. This EEG paste was used because it is easily visible in T1-weighted MR images. The EEG cap was then carefully removed, leaving the electrode positions labelled with EEG paste. An intravenous (iv) catheter was placed in the saphenous vein to induce propofol anaesthesia (mixture of propofol 1 % MCT (Fresenius Kabi AG) and Ringer lactate (1:1), and 1.25 ml ketamine hydrochloride (Ketasol 100^®^, 100 mg/ml, Graeub AG), 1.2–3.6 ml/kg/h). The monkey was placed in lateral decubitus position and insulated with bubble wrap. The monkey’s head was carefully positioned on the side inside the head coil. During the MRI acquisition, the animal’s cardiac pulse frequency and blood saturation rate in oxygen were continuously monitored and the animal was provided with a continuous O_2_ flow. The electrode positions were determined with the EEG paste positions in the MRI space: an Ax FSPGR 3D full head MRI (TE = 3.6 ms, TR = 8,000 ms, ET = 1, flip angle = 10°, acquisition matrix = 240 × 240, 1 excitation) of Mk-EN was acquired on a Discovery MR750 3.0T scanner (GE Medical Systems) with a 32-channel head coil. A total of 312 slices were recorded with a 1.2-mm slice thickness, a 0.6-mm gap between slices and an in-plane resolution of 0.625 × 0.625 mm^2^.

The lead field model was computed with the Cartool software from the CT scan and MRI acquisitions and from the electrode positions. An analytical head model using a manifold of locally adapted spheres to calculate the lead field for each of the 33 electrodes was used (Brunet et al. 2011). The radiuses for the scalp, skull and brain were kept constant (scalp at 100 %, outer skull boundary at 78 % and brain/inner skull boundary at 68 %). Skull stripping was performed with Cartool to obtain the isolated brain. The whole brain, i.e. white and grey matter combined, was used to define a solution space of 3,000 discrete points, because the MRI quality did not provide enough separation between white and grey matter. The relative conductivity of the skull was set to 0.05.

#### Inverse solution

The obtained lead field matrix of Mk-EN was used to compute a LORETA inverse matrix with the Cartool software. A range of 13 Tikhonov regularisations was pre-computed to allow the right amount of regularisation to be selected according to the noise level found in the data.

Left and right median nerve SSEPs from 9 recording sessions in Mk-EN (before the craniotomy) were considered for this analysis. We estimated the CDs (mA/mm^3^) for each source and at each time point. The 100-ms pre-stimulus CD period was used as a baseline. Then, we compared the baseline CDs with the post-stimulus CDs across all the epochs of the different recording sessions using paired *t* tests performed with Matlab (*p* value threshold at 0.05) to assess when stimulus-evoked CDs exceeded the baseline activity (Plomp et al. 2010). The paired samples were the average baseline CD and the evoked CD within each epoch at each time point between 0 and 200 ms after stimulus onset, and for each source point. The *t* values were averaged across each component map and colour-scaled.

### Surgery and craniotomy

The animals of the present study are included in a protocol of cortical lesion of the hand area of M1 (see e.g. Hamadjida et al. 2012; Kaeser et al. 2010, 2011; Wyss et al. 2013). To this aim, animals were trained to perform several manual dexterity tasks (for more details, see Schmidlin et al. 2011) in parallel with SSEPs recordings. The next step in the protocol is to perform a lesion by microinfusion of ibotenic acid at multiple sites within the hand area of M1 (see e.g. Hamadjida et al. 2012; Kaeser et al. 2010, 2011; Liu and Rouiller 1999), requiring consequently a craniotomy to expose the sensorimotor cortex. To evaluate the effect of the craniotomy itself on the SSEPs, a “sham lesion” consisting in the craniotomy alone was first performed in Mk-EN, with the bone flap put back in place.

To perform the craniotomy, the monkey Mk-EN was first sedated with an im injection of ketamine hydrochloride (Ketasol 100^®^, 100 mg/ml, Graeub AG, 10 mg/kg), midazolam hydrochloride (Dormicum^®^, 5 mg/ml, Roche Pharma SA, 0.1 mg/kg) and methadone (Methadone^®^, 10 mg/ml, Streuli Pharma AG, 0.2 mg/kg). The premedication also included atropine (Atropinum sulf^®^, 0.5 mg/ml, Sintetica SA, 0.05 mg/kg, im) to reduce bronchial secretions, the analgesics carprofen (Rimadyl^®^, 50 mg/ml, Pfizer Animal Health, 4 mg/kg, subcutaneous (sc)), the antibiotics ampicillin 10 % (Betamox LA^®^, 150 mg/ml, Arovet SA, 30 mg/kg, sc) and dexamethasone (Dexamethasone^®^, 5 mg/ml, Helvepharm AG, 0.15 mg/kg diluted 1:1 in saline, im) to prevent brain oedema. The surgery itself was performed under sterile conditions. The animal was placed in ventral decubitus position on a heating blanket regulated according to the animal’s rectal temperature, and isolated with bubble wrap. Eye drops (Neosporin^®^, GlaxoSmithKline Inc.) were administrated to prevent exsiccation of the cornea. The intra-operative monitoring was the same as described above for SSEP acquisition (see “Anaesthesia and procedure”) and included in addition body temperature monitoring.

The animal was intubated and put under sevoflurane anaesthesia (Sevorane^®^, Abbott, 2.5 %, in 50 % O_2_ and 50 % air). An iv catheter was placed in the saphenous vein to induce propofol anaesthesia (mixture of propofol 1 % MCT (Fresenius Kabi AG) and Ringer lactate (1:2), 1.8 ml/kg/h) and Ringer lactate infusion (8 ml/kg/h). The monkey’s head was then fixed in a stereotaxic frame (Narishige, Japan) using ear bars coated with lubricating gel (Lidohex^®^, Dr. G. Bichsel AG). The skin was incised along the anteroposterior axis of the head, in the midline. This zone had been locally anaesthetized with several sc injections of lidocaine 1 % (Rapidocain^®^ 1 %, 10 mg/ml, Sintetica SA, 2 ml in total). The muscles were incised and reclined. A craniotomy was performed by drilling a rectangular bone flap (15 mm mediolaterally × 20 mm anteroposteriorally) over the right hemisphere (i.e. contralateral to left median nerve), whose centre was localised 15 mm rostral and 15 mm lateral from the reference point of the stereotaxic frame (half-distance between both ear bars) and with an angle of 30° with respect to the midsagittal plane (Shimazu et al. 2004), giving access to the hand area in the right motor cortex (Fig. 1d). The dura mater was left in place. The bone flap was then repositioned and sutured with 2 stitches, one on the midline anterior part and one on the midline posterior part of the bone flap. To this aim, two small holes were beforehand drilled through the bone flap and two through the skull. A calcium phosphate cement converting to hydroxyapatite (HydroSet Injectable HA Bone Substitute, Stryker^®^; Chow and Takagi 2001; Dickson et al. 2002; Larsson 2006; Van Lieshout et al. 2011) was then applied all around the bone flap and over the stitches to seal the gaps. HydroSet is a synthetic material formed by a sterile white powder (dicalcium phosphate dihydrate, tetracalcium phosphate and trisodium citrate) which has to be mixed with liquid (sodium phosphate, polyvinylpyrrolidone and water) to form a malleable paste. It was mixed and applied with a thin spatula. The muscles and the skin were then sutured. During painful phases (e.g. bone drilling), fentanyl was delivered (Fentanyl Curamed^®^, 0.1 mg/2 ml, Actavis Switzerland AG, 0.1 μg/kg/min diluted 1:1 in saline, iv). Following surgery, the monkey was treated for 9 days with carprofen (Rimadyl^®^, 50 mg/ml, Pfizer Animal Health, 4 mg/kg/day, sc) and ampicillin 10 % (Betamox LA^®^, 150 mg/ml, Arovet SA, 30 mg/kg every second day, sc).

## Results

### Median nerve SSEPs

**Fig. 2:**
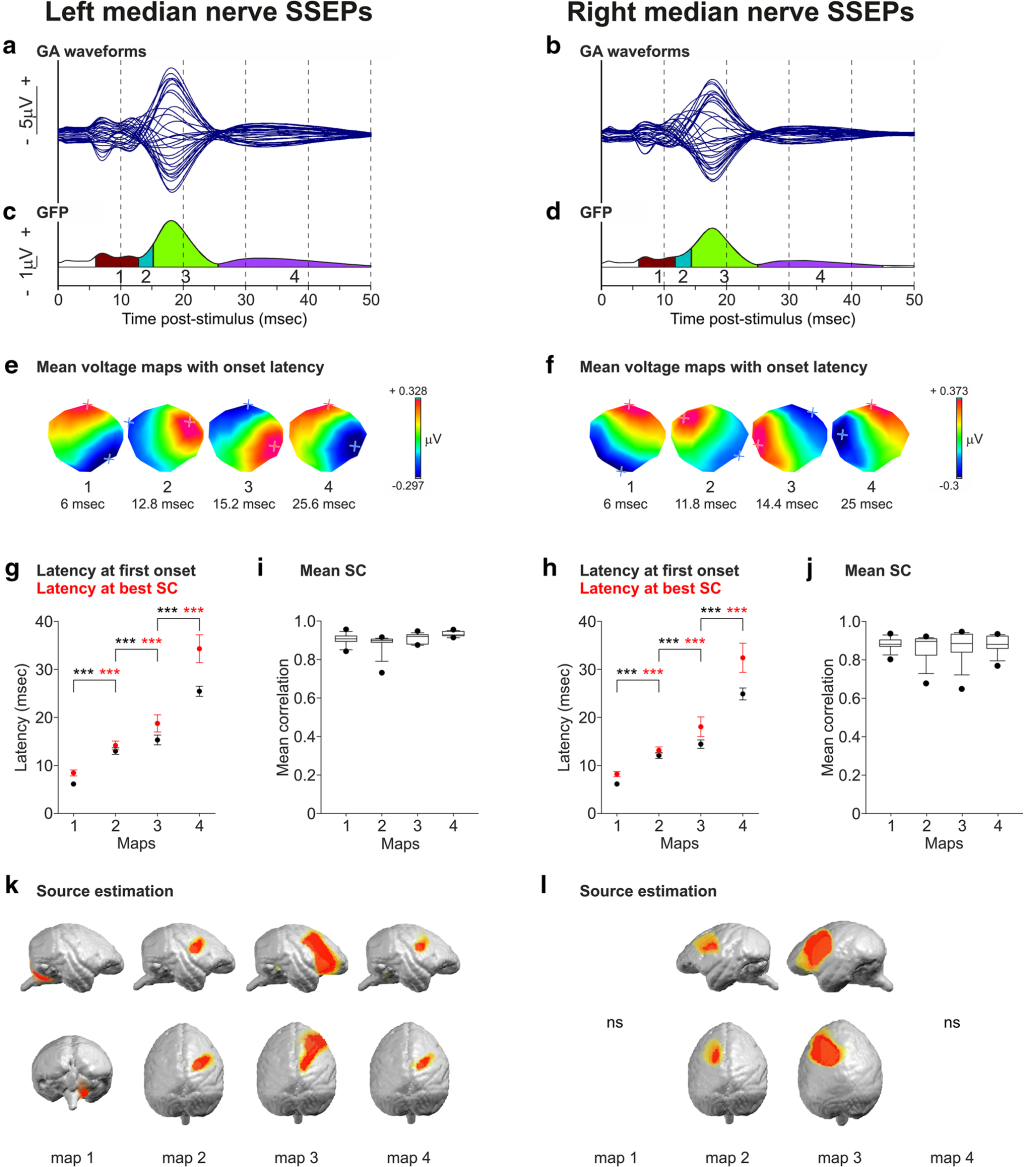
Median nerve SSEPs in Mk-EN. **a**, **b** Overlapped SSEP waveforms at all electrodes after left and right median nerve stimulations (GA of 9 recordings in each case), computed against the average reference, during the first 50 ms following the stimulation. Positive voltages are plotted upward. **c**, **d** Global field power (*GFP*) waveform during the first 50 ms following the stimulation, and temporal extent of the SSEP component maps obtained by cluster analysis. **e**, **f** Colour-scaled mean voltage maps obtained for each cluster shown in **c**, **d**. The *colour* scaling was adapted for each map (positive voltage: *red*, negative voltage: *blue*). *Red* “+” indicates the electrode with the most positive voltage value and *blue* “+” the electrode with the most negative voltage value. The latency at *onset* is indicated for each map. Maps are oriented so that the frontal part points up, the occipital part points down, the left part points left and the right part points right. **g**, **h** Latency at first onset and latency at best spatial correlation (*SC*) for the 4 maps obtained after left and right median nerve stimulations in each of the 9 recording sessions used to compute the GAs shown in **a**, **b**. The mean latency ± SD is shown. 9 unpaired *t* tests and 3 Mann–Whitney *U* rank sum tests when normality tests failed were performed (the *p* value threshold at 0.01 was adapted using Bonferroni correction for the number of electrodes; *** * p* ≤ 0.01) to compare the latencies of pairs of successive maps. **i**, **j** *Box plots* of the mean SC of each map measured for each of the 9 individual recordings used to compute the GAs shown in **a**, **b**. The *bottom* of the *boxes* indicates the 25th percentile, the line within the *boxes* marks the median, and the *top* of the *boxes* indicates the 75th percentile. Whiskers below and above the boxes display the 10th and 90th percentiles, respectively. *Outliers* are represented by *black dots*. **k**, **l** The estimated source localisations obtained with LORETA inverse solution are plotted for each map after left and right median nerve stimulations. *Coloured* areas indicate regions of significant deflection from baseline projected onto Mk-EN’s brain (*t* values are averaged across each component map and colour-scaled, only significant *t* values at *p* < 0.05 are shown, paired *t* tests, *ns* statistically non-significant)

**Fig. 3:**
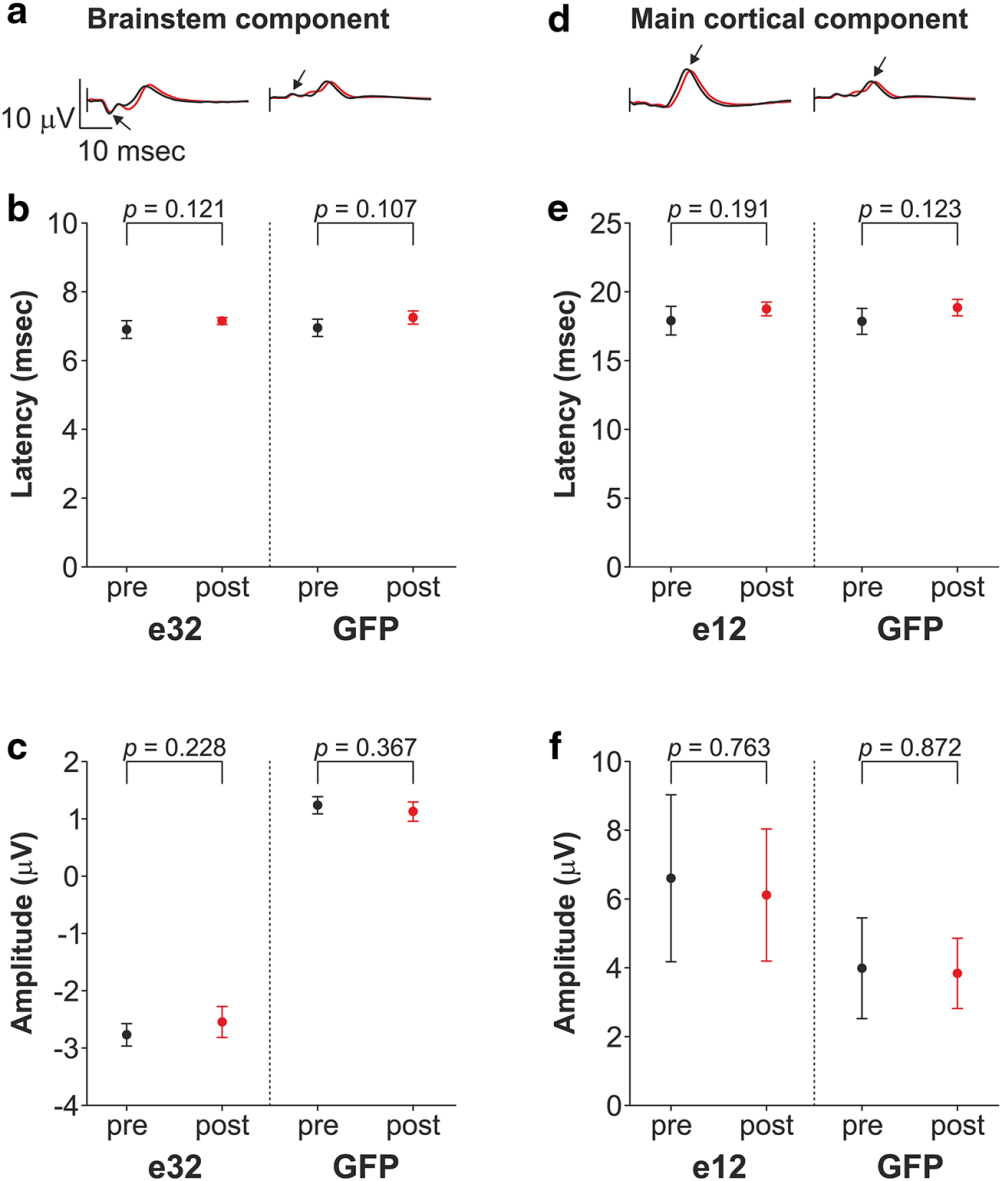
Latencies and amplitudes of left median nerve SSEP components in Mk-EN. **a** Waveform at electrode 32 (e32) (*left*) and GFP waveform (*right*) before (*black*) and after (*red*) craniotomy. The *small black arrow* shows the brainstem component on each waveform. **b** Latency from stimulation onset of the brainstem component measured on e32 and on the GFP waveform before (pre, *black*) and after (post, *red*) craniotomy. **c** Amplitude of the brainstem component measured on e32 and on the GFP waveform. **d** Waveform at electrode 12 (e12) (*left*) and GFP waveform (*right*) before (*black*) and after (*red*) craniotomy. The *small black arrow* shows the main cortical component on each waveform. **e** Latency from stimulation onset of the main cortical component measured on e12 and on the GFP waveform. **f** Amplitude of the main cortical component measured on e12 and on the GFP waveform. The mean ± SD values from 4 pre-craniotomy SSEP (pre) and from 4 post-craniotomy SSEP (post) recordings are plotted for each condition. *p* values obtained with unpaired *t* tests at *p* < 0.01 are indicated. See Fig. 1d for the location of e32 and e12

Electrical stimulation of the left median nerve at the wrist elicited a complex response derived at the scalp (Fig. 2a, b). In Mk-EN, the earliest component was recorded with largest amplitude (mean −2.770 μV, standard deviation (SD) 0.196; mean across 4 pre-craniotomy recording sessions, used for further comparison with 4 post-craniotomy recording sessions, see below “Effect of craniotomy on left median nerve SSEPs”) at 6.9 ms (mean, SD 0.258) (Fig. 3b, c pre) at a contralateral occipital electrode (e32). These spatiotemporal characteristics presumably correspond to the arrival of the afferent volleys in the brainstem (see also results of the inverse solution); this component was consequently called brainstem component. The next major component was recorded with the largest amplitude (mean 6.603 μV, SD 2.428) at 17.9 ms (mean, SD 1.039) (Fig. 3e, f pre) at a contralateral electrode located on the sensorimotor cortex (e12). This component was called here main cortical component.

To characterise the spatiotemporal dynamics of the scalp SSEPs, signals after left and right median nerve stimulations were each averaged from 9 recording sessions regularly distributed at different days over an 11-week period and the GAs were then segmented (GAs of 9 recording sessions performed with Mk-EN, 2 independent clusterings, Fig. 2). The Krzanowski–Lai criterion yielded 4 template maps as the best solution of the K-means cluster analysis (Fig. 2c, d) which explained 97.33 % of the GEV of the sequence of brain activity evoked after left median nerve stimulation and 96.18 % of the GEV of the sequence of brain activity evoked after right median nerve stimulation.

Following left median nerve stimulation, the first SSEP component map lasted from 6 to 12.8 ms after the stimulation (map 1, Fig. 2e) and was characterised by a positive amplitude above the ipsilateral frontal part of the scalp and a strong focal negative amplitude above the contralateral most occipital part of the scalp. The next component map was very short (map 2, from 12.8 to 15.2 ms), with a positive amplitude above the contralateral fronto-parietal cortex and a negative amplitude above the ipsilateral parieto-temporal part of the scalp. Then, the positivity spread towards contralateral parietal electrodes and the negativity became more frontal (map 3, from 15.2 to 25.6 ms). The last component map (map 4, from 25.6 to 50 ms) was characterised by a voltage inversion as compared to map 3. As expected, voltage topographies of SSEPs obtained after left and right median nerve stimulations were essentially mirror images in relation to the anteroposterior axis (Fig. 2e, f**)**. Moreover, the latencies for each component map were highly conserved between both stimulated sides.

A crucial issue is how stable the SSEP signals are in a given monkey across recording sessions from different days since the EEG cap might not be positioned precisely in the same way between recording sessions. The high stability of left median nerve SSEPs was first confirmed by the small SD in amplitude and latency of the brainstem and main cortical components measured on the GFP and, respectively, on e32 and e12, as illustrated in Fig. 3. To address the stability of the component maps across recording sessions, the sequence of 4 templates identified from the GAs was fitted back to the 9 individual recordings (a summary of the raw voltage maps can be found in Online Resource Supplementary Figures 1 and 2). The GEVs of each of the 4 templates of a recording session were added and then averaged across the 9 recording sessions, resulting in remarkably strong mean GEV of 94.64 % (SD 2.26) for left median nerve SSEPs and 93.90 % (SD 0.93) for right median nerve SSEPs. Moreover, the mean latency at first onset and the mean latency at best SC for each component map across the 9 recording sessions were very similar between both right and left median nerve SSEPs and exhibited a very small SD (Fig. 2g, h; Table 1); and the mean SC for each map from the 9 recording sessions was very high for both stimulated sides (Fig. 2i, j; Table 1). Equally important, we observed that the differences in mean latencies at first onset and the differences in mean latencies at best SC between pairs of successive component maps were all highly statistically significant, both after left and right median nerve stimulations (all *p* values ≤10^−3^ for each comparison of two successive map latencies, 9 unpaired *t* tests and 3 Mann–Whitney *U* rank sum tests because normality test failed, Fig. 2g, h; Table 1), indicating that the sequence of component maps was similar across recording sessions. All these observations demonstrate that median nerve SSEPs in macaque monkeys are characterised by a succession of 4 stable component maps highly reproducible across recordings sessions (Fig. 2e, f).

**Table 1:** Fitting parameters of median and tibial nerve SSEPs

	Latency at first onset (ms)		Latency at best SC (ms)		Mean SC		
	Mean	SD	Mean	SD	Mean	SD	Median
Left median nerve SSEPs							
Map 1	6.00	0.00	8.46	0.62	0.90	0.03	0.91
Map 2	13.00	0.68	14.18	0.91	0.88	0.06	0.90
Map 3	15.31	1.02	18.76	1.78	0.91	0.03	0.92
Map 4	25.44	1.04	34.30	2.89	0.93	0.01	0.93
Right median nerve SSEPs							
Map 1	6.00	0.00	8.20	0.52	0.88	0.04	0.88
Map 2	12.09	0.62	13.16	0.71	0.85	0.08	0.90
Map 3	14.44	0.86	18.07	2.05	0.86	0.09	0.89
Map 4	25.05	0.87	32.40	3.04	0.88	0.05	0.88
Left tibial nerve SSEPs							
Map 1	10.91	0.33	12.71	0.96	0.86	0.05	0.85
Map 2	16.53	1.09	22.00	3.21	0.87	0.04	0.88
Map 1	29.31	1.08	40.82	3.92	0.79	0.09	0.83
Right tibial nerve SSEPs							
Map 1	10.94	0.38	12.94	1.08	0.78	0.14	0.82
Map 2	15.76	0.64	21.60	3.32	0.89	0.04	0.89
Map 3	28.97	1.87	40.13	8.25	0.83	0.05	0.84

**Fig. 4:**
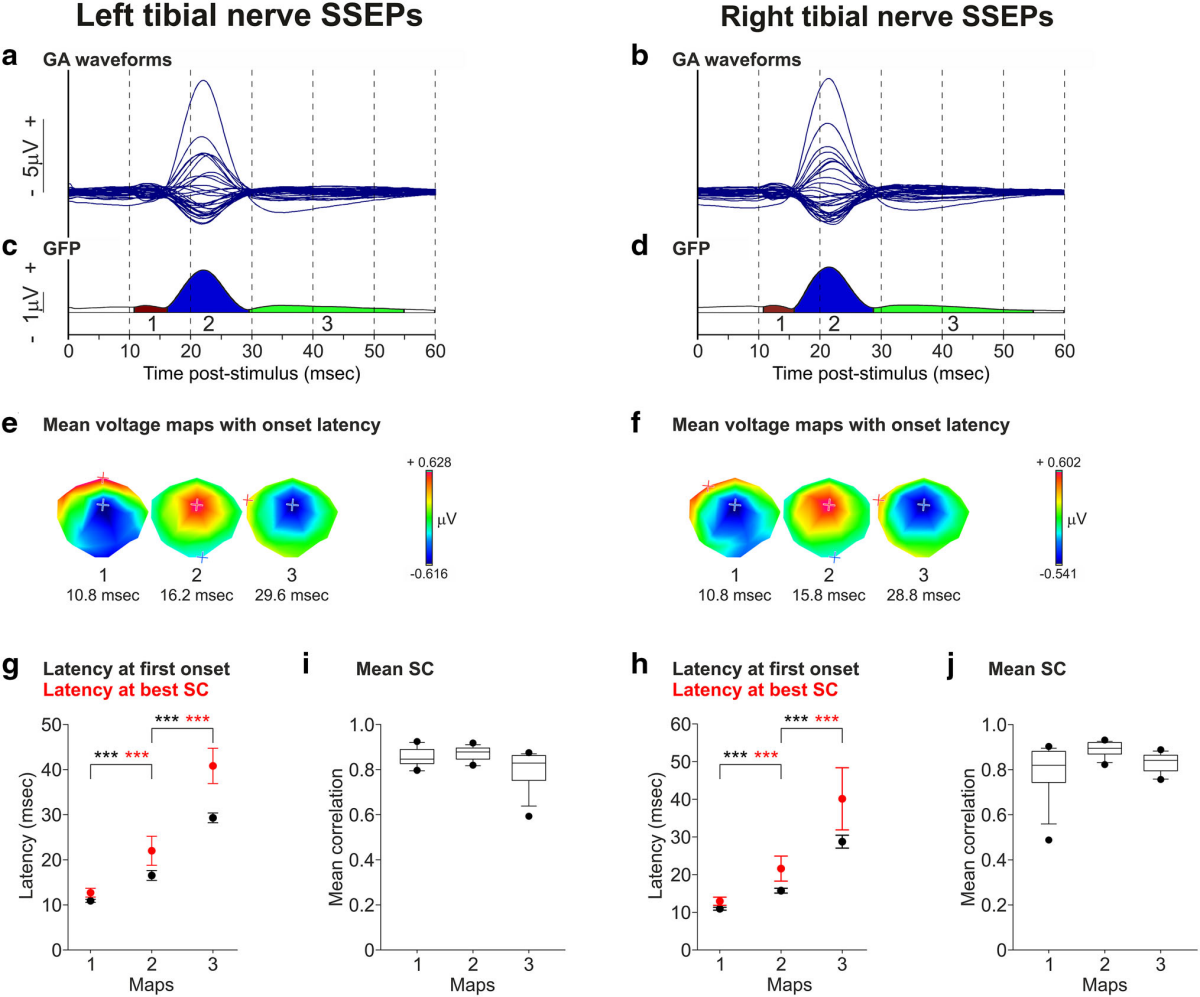
Tibial nerve SSEPs in Mk-EN. **a**, **b** Overlapped SSEP waveforms at all electrodes after left and right tibial nerve stimulations (GA of 9 recordings in each case), during the first 60 ms following the stimulation. **c**, **d** GFP waveform during the first 60 ms following the stimulation, and temporal extent of the SSEP component maps obtained by cluster analysis. **e**, **f** Colour-scaled mean voltage maps obtained for each cluster shown in **c**, **d**. **g**, **h** Latency at first onset and latency at best SC for the 3 maps obtained after left and right tibial nerve stimulations in each of the 9 recording sessions used to compute the GAs shown in **a**, **b**. The mean latency ± SD is shown. 4 unpaired *t* tests and 4 Mann–Whitney *U* rank sum tests when normality tests failed were performed (the *p* value threshold at 0.01 was adapted using Bonferroni correction for the number of electrodes; *** *p* ≤ 0.01) to compare the latencies of pairs of successive maps. **i**, **j** *Box plots* of the mean SC of each map measured for each of the 9 individual recordings used to compute the GAs shown in **a**, **b**. Same conventions as in Fig. 2

Latency at first onset, latency at best spatial correlation (SC) and mean SC resulting from the fitting of the templates (maps) obtained by cluster analysis back to the 9 individual recording sessions of left and right median nerve SSEPs (Fig. 2) and left and right tibial nerve SSEPs (Fig. 4). Values correspond to the mean, SD and median (for mean SC only) across the 9 recording sessions. For reminder, no correlation between the templates and the individual data leads to SC = 0, whereas a whole correlation between them results in SC = 1

The spatiotemporal propagation of activity described by this sequence of component maps seems to correspond well to the expected propagation of sensory evoked processing following median nerve stimulation. During map 1, the strong negative voltage deflections above the contralateral posterior part of the map may correspond to the early processing of the afferent sensory volleys in the dorsal column nuclei in the brainstem. During the 3 following maps, the locations of the strongest voltage values in the contralateral parietal and frontal cortices, first positive during maps 2 and 3, then negative during map 4, may correspond to activation of sensory and motor hand representations. However, no unambiguous statement about the contributing brain areas can be made on the basis of surface topographies alone. Consequently, we used the LORETA distributed, linear inverse solution adapted to Mk-EN’s brain to localise the generators of the observed scalp EEG activities. Figure 2k, l shows the surface representations of the significant intracerebral source estimates during the 4 SSEP maps in response to left and right median nerve stimulations. For left median nerve SSEPs, it confirmed that during map 1, the contralateral dorsal region of the brainstem was active. From maps 2 to 4, activity invaded successively an anterior medial region of the contralateral parietal cortex, then the posterior medial region of the frontal cortex and finally back to the parietal cortex (Fig. 2k). After right median nerve stimulation, similar results were obtained for maps 2 and 3 (Fig. 2l). However, no significant source estimates could be calculated using our algorithm at *p* < 0.05 for maps 1 and 4. This difficulty to localise evoked activity source estimates may be due to morphological differences between the two hemispheres or to the skull altering the positioning of the electrodes above the left and right hemispheres and reducing the signal-to-noise ratio asymmetrically.

### Tibial nerve SSEPs

Although we were interested mainly in the arm representation of the sensorimotor cortex, we also recorded left and right tibial nerve SSEPs (Fig. 4a, b). SSEP signals were averaged from 9 recording sessions regularly distributed over an 11-week period and the GAs were then segmented (GAs of 9 recording sessions performed with Mk-EN, 2 independent clusterings, Fig. 4). The spatiotemporal dynamics of evoked brain activity was summarised by 3 different component maps (Fig. 4e, f) by the K-means cluster analysis (Fig. 4c, d), explaining 97.29 % of the GEV of left tibial nerve SSEPs and 98.04 % of the GEV of right tibial nerve SSEPs. This sequence of 3 templates was then fitted back to the 9 individual recordings. The GEVs of the 3 templates of a recording session were added and then averaged across the 9 recording sessions, yielding a mean GEV of 89.55 % (SD 4.00) for left tibial nerve SSEPs and 90.66 % (SD 3.34) for right tibial nerve SSEPs. Once again, the SD in mean latencies at first onset and in mean latencies at best SC from the 9 recording sessions was small (Fig. 4g, h; Table 1), these latencies were similar between both stimulated sides (Table 1) and the mean SC for each map across the recordings was very high for both left tibial nerve SSEPs and right tibial nerve SSEPs (Fig. 2i, j, Table 1). These findings demonstrate here again that the succession of brain activity components was stable and reproducible across recordings and consequently that the intraindividual variability of tibial nerve SSEP maps was minimal over time.

The differences in mean latencies at first onset and the differences in latencies at best SC between pairs of successive component maps were all highly statistically significant, both after left and right tibial nerve stimulations (all *p* values ≤10^−3^ for each comparison of two successive map latencies, 4 unpaired *t* tests and 4 Mann–Whitney U rank sum tests because normality test failed, Fig. 4g, h; Table 1). Thus, tibial nerve SSEPs in macaque monkeys are remarkably stable across recording sessions (which is also visible in the raw voltage maps in Online Resource Supplementary Figures 3 and 4) and can be characterised by a succession of 3 stable component maps (Fig. 4e, f**)**.

Following left and right tibial nerve stimulations, the SSEPs exhibited first a negative amplitude in the central part of the scalp (fronto-parietal region) and a positive amplitude at the most frontal electrodes (map 1) (Fig. 4e, f). A strong central positive amplitude appeared above midline in the fronto-parietal scalp region during map 2, situated above the expected sensorimotor somatotopic representation of the contralateral leg along the medial longitudinal fissure and reversed during map 3. The similarity of voltage topographies of the SSEPs obtained after left and right tibial nerve stimulations is presumably due to the fact that the leg representation in the sensorimotor cortex is located on either side of the medial longitudinal fissure.

### Interindividual reproducibility of SSEPs

**Fig. 5:**
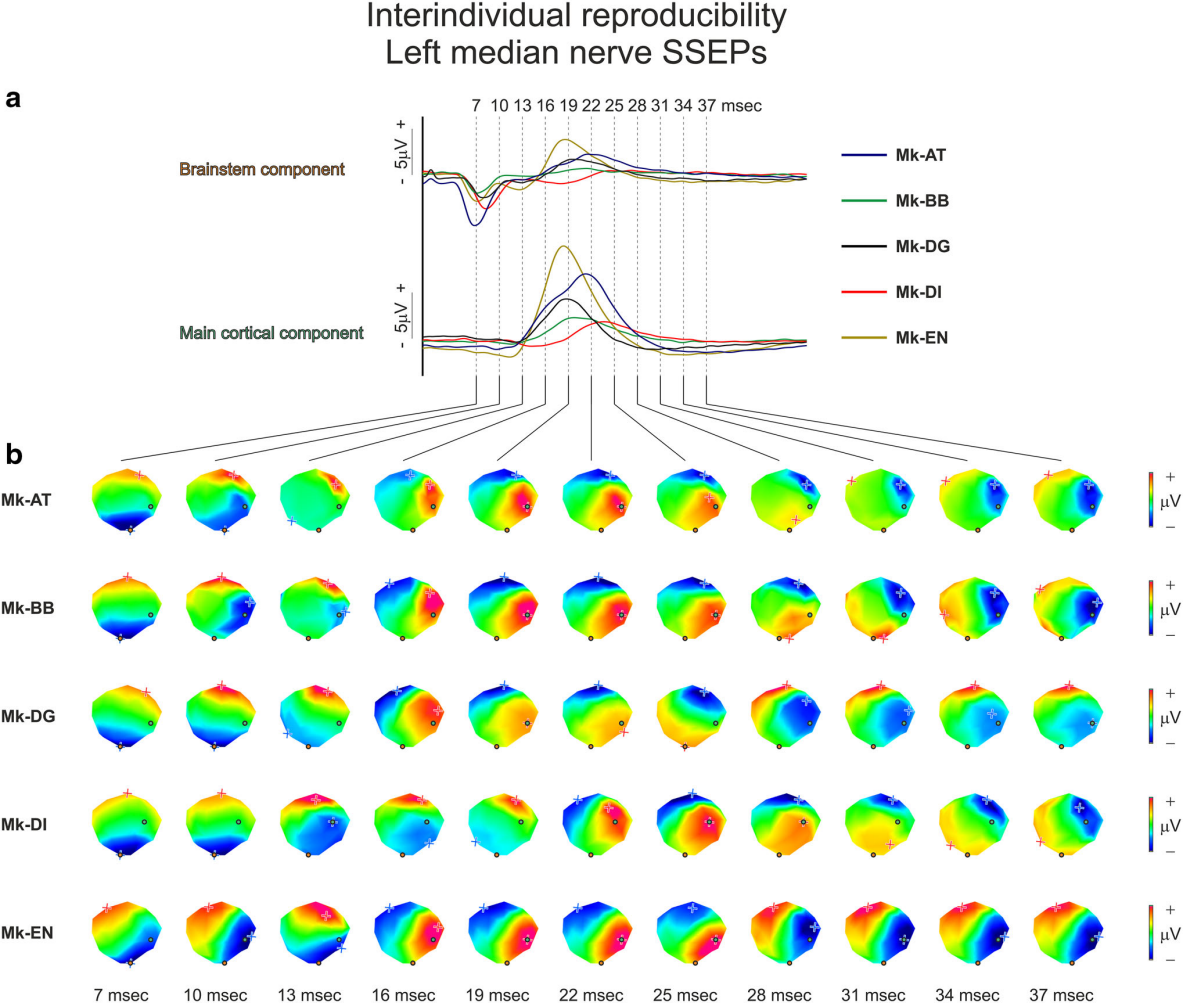
Interindividual reproducibility of left median nerve SSEPs. **a** Brainstem component and main cortical component SSEP waveforms after left median nerve stimulation in five monkeys: Mk-AT (*blue*), Mk-BB (*green*), Mk-DG (*black*), Mk-DI (*red*), and Mk-EN (*yellow*), during the first 50 ms following the stimulation. These data were obtained from 1 recording session in each animal. **b** Colour-scaled voltage maps obtained from 7 to 37 ms post-stimulus, at 3-ms interval. The colour scaling in microvolts is indicated for each animal and was adapted for each map. All the maps were obtained using the same cap model (Mk-EN). The locations of the electrodes where both components were recorded with the largest amplitude are represented on the maps with *orange circles* (brainstem component) and *light green circles* (main cortical component). Note that these locations can vary between animals. Same conventions as in Fig. 2

The results presented in Figs. 2 and 4, acquired in a single animal (Mk-EN), showed high stability across recording sessions. Median nerve and tibial nerve SSEP recordings were also performed in four other monkeys (Mk-AT, Mk-BB, Mk-DG, Mk-DI). A qualitative analysis based on left median nerve SSEPs obtained from 1 recording session in the five animals showed that there were some differences in the relative amplitude and some shifts in latencies of the different SSEP components among the animals (Fig. 5a), i.e. the voltage maps at a given time point may differ slightly across animals. For example, maps from 13 ms in Mk-DI were delayed by 3–6 ms relative to the ones in the other monkeys. More importantly, however, voltage topographies at the scalp were conserved both in terms of spatial configuration and temporal sequence across the five individuals (Fig. 5b). This reproducibility of surface topographies across animals was also true for right median nerve SSEPs and left and right tibial nerve SSEPs (Online Resource Supplementary Figures 5–7).

### Effect of craniotomy on left median nerve SSEPs

A 300-mm^2^ craniotomy was performed over the hand representation in the right sensorimotor cortex followed by bone flap repositioning in Mk-EN. Subsequently, post-craniotomy SSEPs were acquired and compared to pre-craniotomy data, to investigate whether the craniotomy had an impact on the scalp SSEPs. Four post-craniotomy SSEPs in response to left median nerve stimulation (therefore contralateral to the craniotomy) were recorded at regular time points over a 7-week period and compared to 4 pre-craniotomy SSEPs recorded over an 11-week period. No statistically significant differences appeared in the amplitude of the signal before and after the craniotomy when the statistical analysis was performed on each electrode at each time frame (two-tailed unpaired *t* test at *p* < 0.01, Bonferroni corrected for the number of electrodes). Moreover, post-craniotomy waveforms did not show any artefact.

The effect of craniotomy on left median nerve SSEPs was then classically assessed by comparing the absolute amplitude and the latency from the stimulation onset of the brainstem and main cortical components on two electrodes of interest (e12 and e32) and on the GFP (Fig. 3). No statistically significant differences in amplitude and in latency were observed between pre- and post-craniotomy data (all *p* values >0.1 for each comparison of pre- and post-craniotomy data, 8 unpaired *t* tests).

**Fig. 6:**
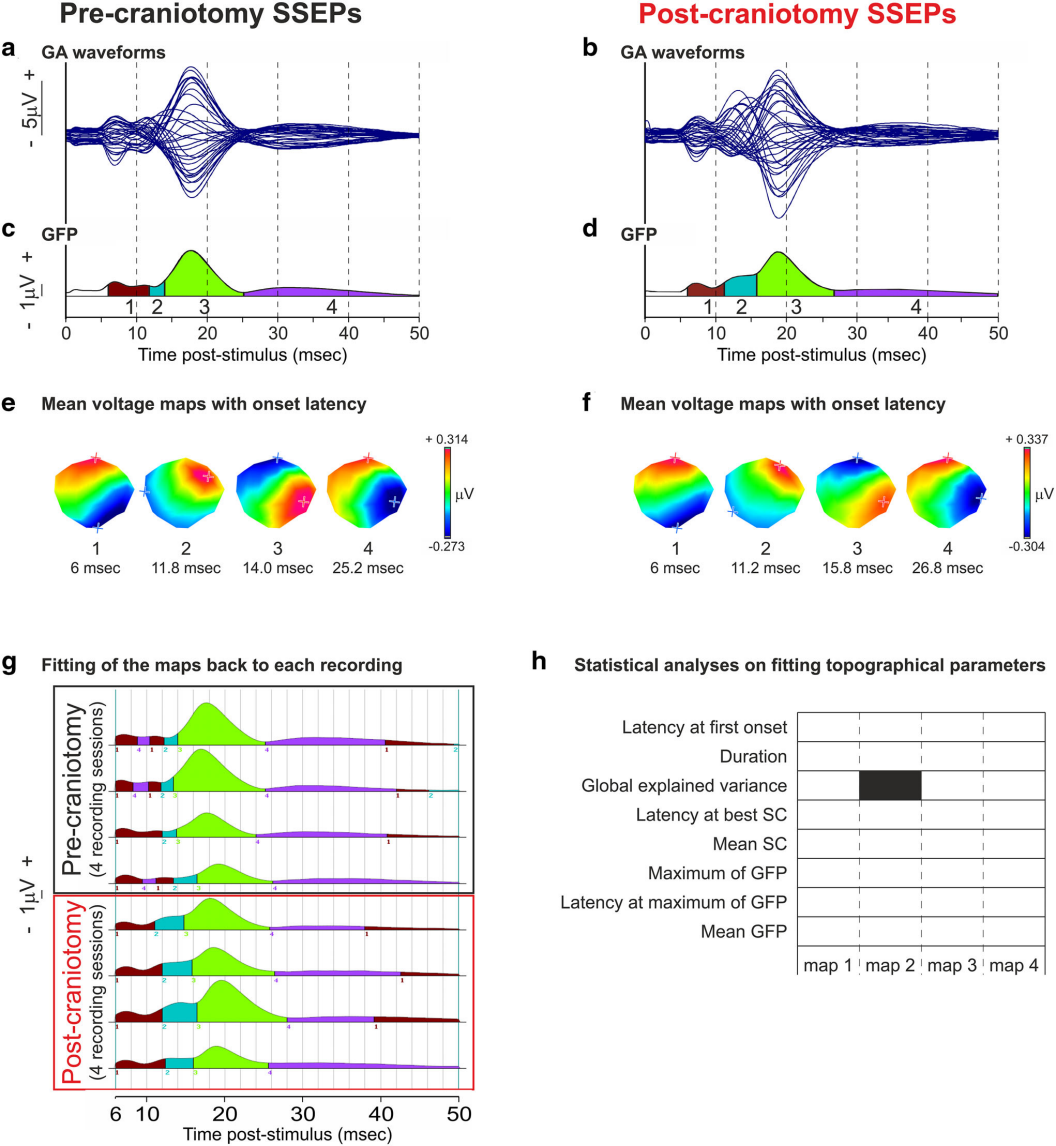
Effect of the craniotomy on the spatiotemporal pattern of left median nerve SSEPs in Mk-EN. **a**, **b** Overlapped SSEP waveforms at all electrodes after left median nerve stimulation obtained before and after craniotomy (GA of 4 recordings in each case). **c**, **d** GFP and temporal extent of the SSEP component maps obtained by cluster analysis. **e**, **f** Colour-scaled mean voltage maps obtained for each cluster shown in **c**, **d**. Same conventions as in Fig. 2. **g**, **h** Fitting process of the 4 distinct clusters obtained by cluster analysis in **c**, **d** back to each of the 8 recordings. **g** GFP waveforms and temporal extent of the 4 different SSEP component maps for each of the 4 pre-craniotomy (*black*) and 4 post-craniotomy (*red*) individual recordings used to compute the GAs shown in **a** and **b**, from 6 to 50 ms following the stimulation. **h** Two-tailed unpaired *t* tests at *p* < 0.01 performed on the fitting results, between the pre- and post-craniotomy recording sessions: *black bars* indicate for each parameter the maps during which *p* values are statistically significant

The effect of craniotomy was also tested using topographical analyses of surface SSEPs (Fig. 6). To this aim, the GA of the 4 pre-craniotomy sessions and the GA of the 4 post-craniotomy sessions used in Fig. 3 were subjected to a common K-means clustering. This segmentation process found the same sequence of 4 SSEP component maps before (Fig. 6c, e) and after (Fig. 6d, f) the surgery with quite similar latencies at first onset, suggesting that craniotomy by itself did not induce major changes in the spatial configuration and the temporal sequence of the component maps. To confirm this result, the 4 maps were fitted back to each of the 4 pre-craniotomy and 4 post-craniotomy recordings (Fig. 6g). Two-tailed unpaired *t* tests were performed to compare 8 topographical parameters (latency at first onset, duration, GEV, latency at best SC, mean SC, maximum of GFP, latency at maximum of GFP and mean GFP) for each component map before and after the craniotomy (Fig. 6h). Despite a seeming increase in cluster 2 duration, no statistically significant differences appeared between both conditions for any map parameter (all *p* values >0.15 for each comparison of pre- and post-craniotomy parameters), except a statistically higher post-craniotomy map 2 GEV (mean 0.101, standard error (SE) 0.005) than pre-craniotomy (mean 0.020, SE 0.007; *p* value 0.0045). This confirmed the absence of any strong adverse effect of craniotomy on our surface EEG and again the intraindividual stability of SSEP recordings over time.

## Discussion

The present study showed that scalp SSEPs can be successfully and reproducibly recorded from a high-density EEG cap in anaesthetized macaque monkeys. Using detailed analyses of waveform components, voltage topographies and source localisation methods, we described the spatiotemporal propagation of SSEPs across the brain and demonstrated the stability of EEG recordings over time and across animals. We also demonstrated that a craniotomy followed by bone flap replacement with calcium phosphate cement suture did not affect the SSEPs in macaque monkeys, confirming that topographical analyses of SSEP are a valid and promising method to assess the reorganisation of the somatosensory network after lesions requiring a craniotomy. This study hence opens up new possibilities for the non-invasive long-term follow-up of cortical reorganisation in macaque monkeys after a cortical lesion or any injury affecting other parts of the central nervous system.

### Intraindividual stability and interindividual reproducibility of the SSEPs

SSEPs recorded over several daily sessions in the same monkey were highly stable in terms of shapes of the waveform components as well as in terms of scalp topographies. This finding is not trivial because it is impossible to position the EEG cap exactly at the same location from one recording session to the next and the impedance of the electrodes also vary between recording sessions. The intraindividual stability of the SSEPs was demonstrated with the fitting process: it is a highly demanding procedure because it tries to allocate the clustering template fitting with the highest SC to the voltage topography of each time frame independently, and in each individual recording independently. Therefore, obtaining a coherent succession of voltage topographies in each recording and conserved across the recordings demonstrates and also proves the high quality and the stability of the SSEP data. Highly reproducible EP recordings from one session to the next were already demonstrated in mice in response to whisker stimulation (Megevand et al. 2008). Between monkeys, the same components were present, although we observed some differences in latencies and amplitudes. These differences in latency and amplitude might be due to intrinsic physiological differences between animals. Latency differences may also be due to anatomical variations, such as the size of the limbs, inducing differences in the length of the nerve tracts from the peripheral receptors to the brain (Chu 1986). Moreover, some variability may result from differences in the EEG caps and electrode types used for the different animals or differences in the signal-to-noise ratios. Nevertheless, voltage topographies were well conserved among the five monkeys. Taken together, the intraindividual stability and the interindividual reproducibility of SSEPs prove the high quality of our data and support the potential of our method of whole-scalp EEG mapping of SSEPs in non-human primates, e.g. in the context of a regular evaluation of the cortical reorganisation following a cortical lesion.

### Spatiotemporal propagation of SSEPs

Here, we described EPs recorded in macaque monkeys as a succession of stable brain states called functional microstates (Lehmann et al. 1987, 2009; Michel et al. 2009). Their presence was demonstrated in many human multichannel EP studies (for reviews, see Brandeis and Lehmann 1986; Michel et al. 1999, 2001; Murray et al. 2008) as well as in rodents in response to whisker stimulation (Megevand et al. 2008, 2009; Quairiaux et al. 2010, 2011). SSEP voltage maps obtained here in monkeys are quite similar to the ones obtained in human with high-resolution EEG mapping of SSEPs (Lascano et al. 2009; van de Wassenberg et al. 2008a) using the same kind of stimulation. The differences in latency observed between both species are due to the longer human sensory pathways as compared to the ones in macaque monkeys. As reported here, scalp topographies of right and left median nerve SSEPs in human are mirror images in relation to the anteroposterior axis (Lascano et al. 2009), which could be of importance to study the effects of unilateral brain lesions (Quairiaux et al. 2010).

### Source imaging

Based on anatomical knowledge, one could localise the main deep generators of the median nerve SSEPs recorded at occipital electrodes to the brainstem and at contralateral parietal and frontal electrodes to the underneath cortical areas, as already demonstrated in human (Finke et al. 2013; He et al. 2002) and in accordance with a response of the dorsal column nuclei following an electrical stimulation of the median nerve at the wrist in macaque monkeys (Moller et al. 1989) and close to the expected location of the somatosensory representation of the hand in macaque monkeys (Nelson et al. 1980). Moreover, as expected, the scalp SSEP response after tibial nerve stimulation was medial to the one after median nerve stimulation. Nevertheless due to the volume conduction problem and to the distance to the generators, the validity and the spatial resolution of such observations are limited. Classically, the localisation of generators of brain activity was investigated in monkeys using invasive recordings on the surface of or within the cortex (for a review, see e.g. Allison et al. 1991a). Here, we used a distributed source localisation method on the scalp EEG of macaque monkeys. Distributed source localisation methods have been successfully used in many previous human experimental studies (for reviews, see e.g. He et al. 2011; Michel et al. 2001, 2004; Michel and He 2011) and clinical studies in the context of the localisation of epileptic foci (see e.g. Plummer et al. 2010). Our results could be of interest for future lesion-induced plasticity experiments. To the best of our knowledge, LORETA source analyses based on high-density EEG have been used only in two other studies with monkeys (Fontanarosa et al. 2004; Gil-da-Costa et al. 2013). The present study confirms the feasibility of recording scalp EEGs from a high-density electrode array in non-human primates and of localising the cortical generators of EPs with LORETA. Moreover, our study on SSEPs is original from several points of view: first of all, we developed for the first time scalp EEG recordings of SSEPs in adult *M. fascicularis* with a large number of scalp electrodes. Such whole-scalp recordings allow to record large-scale neuronal networks and their reorganisation following a disruption. We can assume that we recorded EEG with a higher density of scalp electrodes as compared to 22 electrodes (Gil-da-Costa et al. 2013) and 32 electrodes (Fontanarosa et al. 2004) in adult *M. mulatta*, that have a larger head than *M. fascicularis* (Hamada et al. 2006), although the size and weight of the animals involved in both studies were not mentioned. Then, we demonstrated that SSEP signals were topographically stable over time in the same animal and across several animals, which is required to prove the validity of our method of EEG mapping of SSEPs in macaque monkeys. To this aim, we introduced for the first time a cluster analysis of monkey SSEPs with detailed statistical analyses of the voltage topographies which are reference-independent instead of waveforms at selected electrodes (Geselowitz 1998; Michel et al. 2004; Murray et al. 2008). Equally important, we developed and applied a LORETA inverse solution to SSEP signals in macaque monkeys. Last but not least, we demonstrated that a replaced, sutured and cemented bone flap following a craniotomy had a negligible effect on the recorded EEG signals in macaque monkeys.

### Effect of craniotomy on scalp SSEPs

EEGs in patients with a skull defect or a skull lesion are characterised by a “breach rhythm” that is not suppressed in all cases after skull reconstruction (Brigo et al. 2011; Cobb et al. 1979; Cobb and Sears 1960; van Doorn and Cherian 2008). The breach rhythm signals are a mu-like activity and exhibit a higher overall power as compared to normal scalp spontaneous EEGs and EPs (Lee et al. 2010a; Pfurtscheller et al. 1982; Tatum et al. 2011; Voytek et al. 2010). These signals reflect probably the reduction of the filtering and of high resistive properties normally exerted by the intact skull, resulting in a higher current flow from the brain to the scalp (Brigo et al. 2011; Chauveau et al. 2004). The amplitude of these signals depends among others on the distance between the recording electrode and the hole, on the hole size and conductivity, and on the orientation and location of the source in relation to the skull defect (Benar and Gotman 2002; Chauveau et al. 2004; Flemming et al. 2005; Heasman et al. 2002; Li et al. 2007; Oostenveld and Oostendorp 2002). Thus, the signal distortion is variable for the different components of EPs, depending on the location of the involved source in relation to the skull defect (Flemming et al. 2005). It is important to take this EEG alteration into account to achieve an accurate EEG source localisation (Benar and Gotman 2002; Chauveau et al. 2004; Chen et al. 2010; Heasman et al. 2002; Oostenveld and Oostendorp 2002). In addition to these breach rhythms, a recent publication (Suzuki et al. 2012) reports that a craniotomy may induce artifactual glial activation (Xu et al. 2007) due to mechanical stimulation (Davalos et al. 2005), and cortical inflammation due to inflammatory blood cell leakage from damaged vessels.

The effect of the craniotomy on SSEP signals was assessed by two complementary approaches: the classical analysis gives information about differences in the signal amplitude and latency between two situations, whereas the topographical analysis illustrates changes in the electrical field distribution (Astle et al. 2009). Classical analyses showed no statistically significant effect of craniotomy. No differences were observed in the topographical parameters studied here except the GEV of map 2 that was higher in post-craniotomy recordings. As reminder, the GEV is the percentage of the data variance explained by a given voltage topography and therefore should indicate the importance of a given map (Brunet 1996, Cartool Reference Guide; Koban et al. 2012). It means that the significance of map 2 was higher after than before the craniotomy but the topography, the latency at first onset, the duration, the SC and the GFP of this map were not affected by the surgery, i.e. the syntax (temporal sequence and duration) of the SSEP component maps did not significantly change after craniotomy.

The observation that craniotomy followed by bone flap repositioning had a negligible effect on the SSEP signals in macaque monkeys shows the beneficial effect of using calcium phosphate cement to plug the entire perimeter of the bone flap. Such hydroxyapatite bone substitutes present several advantages: they can be resorbed and then replaced by natural bone under physiological conditions (osteoconductive properties) because they support bone proliferation; they are biocompatible; they have a long working time (time from start of mixing, allowing to manipulate the cement) and a short setting time; they can be set in a wet field environment; they do not release any heat during setting (isothermic properties) and finally they can be applied by simple injection (Adams et al. 2011; Clarkin et al. 2009a, b; Hannink et al. 2008). Our results contrast with the “breach rhythm” reported by Cobb et al. (1979) in some patients after skull reconstruction following craniotomy. Nevertheless, we should keep in mind that immediately after a surgery breach rhythms can be absent and develop progressively instead (Pfurtscheller et al. 1982). In a recent study using median nerve and tibial nerve SSEPs to evaluate the extent of surgical decompression in children affected by Chiari type 1 malformation (Chen et al. 2012), the bone flap was not replaced at the end of the surgery (craniectomy) in some patients whereas it was (craniotomy) in the others. Nevertheless, there was no difference in the pattern of decrease of SSEP latency during the surgery between both groups of subjects, meaning that the replacement of the bone did not influence SSEPs and therefore that the EP latency decrease observed during the surgery was the result of the decompression on its own (by craniectomy and durotomy). This finding goes in the same direction as the results obtained here in macaque monkeys. The demonstration of negligible effect of the craniotomy on SSEPs is important in the context of a future unilateral lesion of M1 requiring a craniotomy, to distinguish the possible modulations generated by the craniotomy from the consequences of the lesion itself on SSEP responses.

### Future perspectives

Based on previous studies (Bazley et al. 2011; Hu et al. 2011), SSEPs are expected to help to investigate the post-lesional cortical reorganisation of neuronal networks, especially to highlight which areas of the monkey’s brain may take over the functions of M1 affected by the lesion. From a clinical point of view, we also hope that post-lesional modifications in SSEP signals will help us to predict the level of recovery after the lesion (Carter and Butt 2005; Feys et al. 2000; Lee et al. 2010b, c; Su et al. 2010; Tzvetanov et al. 2005; Tzvetanov and Rousseff 2005; Zhang et al. 2011).

To sum up, the present study demonstrated the feasibility of high-density scalp SSEP recordings during the pre- and post-lesional follow-up of cortical activity in macaque monkeys. Not only is EEG relatively inexpensive and non-invasive as compared to other imaging techniques, allowing repeated acquisitions in the same animal, but it also allows to study cortical reorganisation at the whole-brain level and with high temporal resolution, i.e. in the ms time scales, a temporal resolution consistent with the speed of information processing (Michel and Murray 2012; Nunez 1993). Therefore, SSEPs might give additional information to ICMS or LSI approaches from a temporal and large-scale networks point of view and will help to unravel the different mechanisms involved in cortical reorganisation following a brain lesion. Future perspectives will include EEG recordings of SSEPs following a unilateral permanent lesion of the hand representation in M1 in macaque monkeys, and subsequently the application of this EEG method in awake monkeys.

## Electronic supplementary material

Below is the link to the electronic supplementary material.

Supplementary material 1 (PDF 13 kb)

Supplementary material 2 (TIFF 25506 kb)

Supplementary material 3 (TIFF 25901 kb)

Supplementary material 4 (TIFF 26735 kb)

Supplementary material 5 (TIFF 27497 kb)

Supplementary material 6 (TIFF 15362 kb)

Supplementary material 7 (TIFF 15217 kb)

Supplementary material 8 (TIFF 15370 kb)

## Abbreviations

CD: Current density
EEG: Electroencephalography
EPs: Evoked potentials
GA: Grand average
GEV: Global explained variance
GFP: Global field power
ICMS: Intracortical microstimulation
im: Intramuscular
iv: Intravenous
LORETA: Low-resolution electromagnetic tomography
LSI: Laser speckle imaging
M1: Primary motor cortex
MRI: Magnetic resonance imaging
S1: Primary somatosensory cortex
SC: Spatial correlation
sc: Subcutaneous
SD: Standard deviation
SE: Standard error
SSEPs: Somatosensory evoked potentials
