# Supplementary material for: Whole-scalp EEG mapping of somatosensory evoked potentials in macaque monkeys
Source: Brain Struct Funct. 2014 May 4;220(4):2121–42. doi: 10.1007/s00429-014-0776-y (PMC4495608; doi:10.1007/s00429-014-0776-y)
Supplement: Supplementary file 1 — Supplementary material 1 (PDF 13 kb) [file 429_2014_776_MOESM1_ESM.pdf]

Whole-scalp EEG mapping of somatosensory evoked potentials in macaque monkeys

Gindrat Anne-Dominique[1,*], Quairiaux Charles[2,3,*], Britz Juliane[3,4], Brunet Denis[3,4], Lanz Florian[1], Michel Christoph M.[3,4,&] and Rouiller Eric M.[1,&]

[1] Domain of Physiology, Department of Medicine, Faculty of Sciences and Fribourg Center for Cognition, University of Fribourg, Chemin du Musée 5, CH-1700 Fribourg, Switzerland.
[2] Faculty of Medicine, Department of Fundamental Neurosciences, University of Geneva, rue Michel-Servet 1, CH-1206 Geneva, Switzerland
[3] Functional Brain Mapping Laboratory, Departments of Clinical and Fundamental Neurosciences, University Medical School, rue Michel-Servet 1, CH-1206 Geneva, Switzerland
[4] EEG Brain Mapping Core, Center for Biomedical Imaging (CIBM), University Hospital and University of Geneva, CH-1211 Geneva, Switzerland

* Equal first authorship
& Equal senior authorship

Correspondence:

Prof. Eric M. Rouiller (same address as above): eric.rouiller@unifr.ch, phone: + 41 26 300 86 09

or

Prof. C. Michel (same address as above): Christoph.Michel@unige.ch, phone: +41 22 379 54 57

Supplementary Fig. 1 Intraindividual stability of left median nerve SSEPs in Mk-EN. Colour-scaled voltage maps of each of the 9 individual SSEP recording sessions from different days used to compute the GA shown in Figure 2A, obtained from 7 to 40 msec post-stimulus at 3-msec interval. Same conventions as in Figure 2

Supplementary Fig. 2 Intraindividual stability of right median nerve SSEPs in Mk-EN. Colour-scaled voltage maps of each of the 9 individual SSEP recording sessions from different days used to compute the GA shown in Figure 2B, obtained from 7 to 40 msec post-stimulus at 3-msec interval. Same conventions as in Figure 2

Supplementary Fig. 3 Intraindividual stability of left tibial nerve SSEPs in Mk-EN. Colour-scaled voltage maps of each of the 9 individual SSEP recording sessions from different days used to compute the GA shown in Figure 4A, obtained from 12 to 45 msec post-stimulus at 3-msec interval. Same conventions as in Figure 2

Supplementary Fig. 4 Intraindividual stability of right tibial nerve SSEPs in Mk-EN. Colour-scaled voltage maps of each of the 9 individual SSEP recording sessions from different days used to compute the GA shown in Figure 4B, obtained from 12 to 45 msec post-stimulus at 3-msec interval. Same conventions as in Figure 2

Supplementary Fig. 5 Interindividual reproducibility of right median nerve SSEPs. (A) Brainstem component and main cortical component SSEP waveforms after right median nerve stimulation in 5 monkeys: Mk-AT, Mk-BB, Mk,-DG, Mk-DI, and Mk-EN, during the first 50 msec following the stimulation. These data were obtained from 1 recording session in each animal. **(B)** Colour-scaled voltage maps obtained from 7 to 37 msec post-stimulus, at 3-msec interval. Same conventions as in Figures 2 and 5

Supplementary Fig. 6 Interindividual reproducibility of left tibial nerve SSEPs. (A) Brainstem component and main cortical component SSEP waveforms after left tibial nerve stimulation in 5 monkeys: Mk-AT, Mk-BB, Mk,-DG, Mk-DI, and Mk-EN, during the first 60 msec following the stimulation. These data were obtained from 1 recording session in each animal. **(B)** Colour-scaled voltage maps obtained from 12 to 42 msec post-stimulus, at 3-msec interval. Same conventions as in Figures 2 and 5

Supplementary Fig. 7 Interindividual reproducibility of right tibial nerve SSEPs. (A) Brainstem component and main cortical component SSEP waveforms after right tibial nerve stimulation in 5 monkeys: Mk-AT, Mk-BB, Mk,-DG, Mk-DI, and Mk-EN, during the first 60 msec following the stimulation. These data were obtained from 1 recording session in each animal. **(B)** Colour-scaled voltage maps obtained from 12 to 42 msec post-stimulus, at 3-msec interval. Same conventions as in Figures 2 and 5
